# Supplementary material for: Large contribution of fossil-derived components to aqueous secondary organic aerosols in China
Source: Nat Commun. 2022 Aug 31;13:5115. doi: 10.1038/s41467-022-32863-3 (PMC9433442; doi:10.1038/s41467-022-32863-3)
Supplement: Supplementary file 1 — Supplementary Information [file 41467_2022_32863_MOESM1_ESM.pdf]

Supporting Information for

Large contribution of fossil-derived components to aqueous secondary organic aerosols in China

Buqing Xu, Gan Zhang, Örjan Gustafsson, Kimitaka Kawamura, Jun Li, August Andersson, Srinivas Bikkina, Bhagawati Kunwar, Ambarish Pokhrel, Guangcai Zhong, Shizhen Zhao, Jing Li, Chen Huang, Zhineng Cheng, Sanyuan Zhu, Pingan Peng, and Guoying Sheng

To whom correspondence should be addressed. Email: G.Z.(zhanggan@gig.ac.cn) and Ö.G. (orjan.gustafsson@aces.su.se)

This PDF file includes:

1. Supplementary Methods (Method 1–4)

2. Supplementary Figures (Figure 1–10)

3. Supplementary Tables (Table 1–10)

4. Supplementary References

Supplementary Method 1. Methodology of Supporting Chemical Composition

Mass concentration of $PM_{2.5}$ was ascertained gravimetrically by weighing the full filters (with a precision of 0.1 mg) before and after the sampling. Prior to their weighing, all filters were conditioned at a relative humidity of 45 ± 5 % and temperature of 25 ± 1°C for 24 hrs. Organic carbon (OC) and elemental carbon (EC) were measured on a 1.5 cm punch from each filter by an OC/EC analyzer (Sunset Laboratory, Inc., USA) with a FID detector, run by NIOSH thermal-optical transmittance (TOT) standard method. In addition, a part of each filter (4.5 cm^2) was extracted by Milli-Q water (10ml × 3 times) under ultrasonication and passed through a 0.22 μm PTFE filter head (Jinteng, China). Half of the extraction was analyzed for water-soluble organic carbon (WSOC) and water-soluble organic nitrogen (WSON) by a total organic carbon analyzer (TOC-VCPH, Shimadzu). The other half of the extraction was analyzed for water soluble inorganic constituents (Na^+, NH_4^+, K^+, Mg^{2+}, Ca^{2+}, Cl^-, NO_3^-, and SO_4^{2-}) and was analyzed on an ion chromatography (761 Compact IC, Metrohm, Switzerland)[1].

For organic molecular tracers (secondary organic aerosol tracers and Levoglucosan), a portion of the aerosol sample (1.9 - 9.7 cm^2) was extracted with a dichloromethane and methanol mixture (2:1) by ultrasonic agitation (10 mL × 3 times). The secondary organic aerosol (SOA) tracers analyzed in this study include isoprene SOA tracers (2-methylglyceric acid, 2-methylthreitol, 2-methylerythitol, cis-2-methy-1,3,4-trihydroxy-1-butene, 3-methyl-2,3,4-trihydroxy-1-butene, trans-2-methyl-1,3,4-trihydroxy-1-butene) and α/β monoterpene SOA tracers (3-hydroxyglutaric acid, pinonic acid, pinic acid). The solvent extracts were filtered through quartz wool packed in a Pasteur pipette, concentrated by rotary evaporator, and blown down to dryness with pure nitrogen gas into a 1.5 mL glass vial. To this vial, we added 50 μL of silylation reagent containing a mixture of N,Obis(trimethylsilyl) trifluoroacetamide (BSTFA) with 1% TMCS (trimethylchlorosilane) and 10 μL of pyridine and heated at 70 °C for 3 h. After derivatization, 60 μL of C_{13} n-alkane (1.43 ng μL^{-1}) was added as an internal standard. Gas chromatography/mass spectrometry (GC/MS) analyses were performed for the derivatized extracts using an Agilent 6890 GC coupled to Agilent 5973 mass-

selective detector. We assessed the mass concentrations of organic tracers by using the analytical protocol described in earlier publications[2, 3]. The concentrations of organic tracers, inorganic ions, WSOC and OC, EC reported here are all corrected for the field blanks.

Supplementary Method 2. Estimation of Aerosol Liquid Water (ALW) Content

The effect of inorganic ions on ALW was estimated using a thermodynamic equilibrium model for the K^+–Ca^{2+}–Mg^{2+}– NH_4^+–Na^+–SO_4^{2-} –NO_3^-–Cl^-–H_2O aerosol system, ISORROPIA-II[4]. It can analyze gas-liquid -particulate steady phase for inorganic compounds by running the reverse mode iteratively based on ion concentration, relative humidity and temperature data. For our analysis at Heshan, the inputs of ISORROPIA-II are the inorganic ions measured by IC, RH and temperature from the hourly monitor by Heshan Atmospheric Environmental Monitoring Superstation.

We apply k-Kohler theory with the Zdanovskii−Stokes−Robinson (ZSR) mixing Rule to describe the hygroscopic growth of organic compounds in aerosols using Eq. S1 as follows[5]:

$$V_{w,o} = V_o \times k_{org} \times (a_w/1- a_w) \quad \textbf{Eq. S1}$$

Where $V_{w,o}$ and V_o represent ALW and organic compound volumes, respectively, k_{org} is the organic component hygroscopicity (dimensionless), and a_w is the water activity (dimensionless). Here, organic matter (OM) is calculated by organic carbon (OC) with a ratio of 1.73 based on an earlier study in Heshan[6], V_o is calculated by an assumed organic density of 1.4 g/cm^3 for OM[5] and 1.9 g/cm^3 for oxalic acid[7]. We apply k_{org} values of 0.11 for organic aerosols at continental outflow[5] and 0.48 for oxalic acid[7]. We assume a_w is equivalent to RH for the sake of simplicity because of a lack of particle diameter data.

Supplementary Method 3. Calculation of source apportionment results from raw radiocarbon data

1. Radiocarbon blank characterization and correction

A pair of processing standards across a range of sample size (25–250 μg) with a modern (n-docosane; F_m = 1.0524 ± 0.0058) or dead ^{14}C composition (phthalic acid; F_m = 0.0028 ± 0.0001) were added and analyzed alongside the full sample processing to calculate the negative/positive exogenous or nonspecific background carbon (C_{ex}). Isotopic offsets from the original F_m values of the modern n-docosane gave the dead-carbon contamination (DCC; with assumed Fm_{ex} = 0), whereas the modern-carbon contamination (MCC; with assumed Fm_{ex} = 1) was determined from the offset from the original F_m values for the phthalic acid with dead ^{14}C composition. The DCC and MCC were respectively calculated by the following equation:

$$Fm_{\text{sample}} \times C_{\text{sample}} = Fm_{\text{measure}} \times C_{\text{measure}} - Fm_{\text{ex}} \times C_{\text{ex}} \qquad \textbf{Eq. S2}$$

where $C_{measure}$ is the manometrically quantified total carbon mass, C_{sample} is equivalent to $C_{measured}$-C_{ex}, and C_{ex} is DCC+MCC.

Then, the calculated Fm_{ex} and C_{ex} were used for blank correction of the samples, using the following equation:

$$Fm_{\text{corr}} = \frac{Fm_{\text{measure}} \times C_{\text{measure}} - Fm_{\text{ex}} \times C_{\text{ex}}}{C_{\text{measure}} - C_{\text{ex}}} \qquad \textbf{Eq. S3}$$

where $Fm_{measure}$ being the result reported from AMS facility, Fm_{corr} is the radiocarbon signals of sample with blank correction, $C_{measure}$ is the manometrically quantified carbon mass for the uncorrected sample.

2. **Correction for the carbon contribution of 1-butanol**

After blanks correction, an isotopic mass balance approach was adopted to correct the carbon contribution of butanol groups (-C_4H_9) introduced in the derivatization of diacids, where appropriate:

$$Fm_{\text{DABE}} = f_{\text{Diacid}} \times Fm_{\text{Diacid}} + f_{\text{BuOH}} \times Fm_{\text{BuOH}} \qquad \textbf{Eq. S4}$$

where f_{Diacid} and f_{BuOH} are fractions of carbon in the esters derived from diacids and 1-butanol. For example, f_{Diacid} and f_{BuOH} for oxalic acid is 0.2 and 0.8, respectively.

The f_{Diacid} of each dicarboxylic acid, oxocarboxylic acid, and α–dicarbonyls in the 1-butanol derived derivatives can be found elsewhere[8]. The $Fm^{14}C$ value of individual diacids (Fm_{Diacid}) was then calculated based on the values of the derivative (Fm_{DABE}, i.e., the Fm_{corr} in Eq. S3) and 1-butanol (Fm_{BuOH})that were measured by AMS. Here, Fm_{BuOH} is repeated measured five times, and the average F_m values is 0.0029 ± 0.0001.

3. Error assessment

The error of the Fm_{DABE} values (i.e., Fm_{corr} in Eq. S3) is calculated using error propagation:[9, 10]

$$\sigma_{\mathrm{DABE}} = \sqrt{\sum_{i=1}^{n}(\frac{\partial Fm}{\partial x_i})\sigma_{x_i}{}^2} \qquad \textbf{Eq. S5}$$

Where σ_{x_i} include the uncertainty for AMS uncertainty of Fm measured ($\sigma_{Fm_{\mathrm{measure}}}$), the uncertainty for Fm_{ex} ($\sigma_{Fm_{\mathrm{ex}}}$), the uncertainty for carbon masses of C_{measured} and C_{ex} ($\sigma_{C_{\mathrm{measure}}}$ and $\sigma_{C_{\mathrm{ex}}}$, respectively).

Based on the calculated σ_{DABE}, The error of the Fm_{Diacid} (σ_{Diacid}) is calculated using the following equation:

$$\sigma_{\mathrm{diacid}}{}^2 = \left(\frac{1}{f_{\mathrm{Diacid}}}\right)^2 \times \sigma_{\mathrm{DABE}}{}^2 + \left(\frac{1}{f_{\mathrm{BuOH}}}\right)^2 \times \sigma_{\mathrm{BuOH}}{}^2 \qquad \textbf{Eq. S6}$$

where the σ_{BuOH} is the error of Fm_{BuOH} from repeated AMS analysis, the f_{Diacid} and f_{BuOH} are the fractions of carbon in the esters derived from original compound carbon and derivatizing reagent (1–butanol), respectively.

4. Conversion of the F_m value to the fraction of non-fossil source

To correct excess ^{14}C from nuclear bomb tests in the 1950s and 1960s, Fm values were converted to the fraction of non-fossil source ($f_{\mathrm{bio/bb}}$) as follows:

$$f_{\mathrm{bio/bb}} = \frac{F_m}{F_{m,\mathrm{ref}}} \qquad \textbf{Eq. S7}$$

Where the $F_{m,\mathrm{ref}}$ is a reference value of F_m for contemporary carbon sources including biogenic emissions and biomass burning ($F_{m,\mathrm{bio}}$ and $F_{m,\mathrm{bb}}$, respectively). The values of $F_{m,\mathrm{bio}}$ and $F_{m,\mathrm{bb}}$ is parameterized following previous protocol[11]. $F_{m,\mathrm{bio}}$ is obtained from global contemporary $^{14}CO_2$ in year 2017–2018 at two representative background $\Delta^{14}CO_2$ observation station located in northern Hemisphere (Jungfraujoch;

$\Delta^{14}CO_2$=5.3‰; https://data.icos-cp.eu) and Southern Hemisphere (Wellington; $\Delta^{14}CO_2$ =14.8‰; https://www.gns.cri.nz), respectively[12]. The value of $F_{m,\ \mathrm{bb}}$ is higher than $F_{m,\ \mathrm{bio}}$ because it is reflecting the $\Delta^{14}C$ of biomass that has accumulated over the decades-to-century-long life span of trees. For East Asia, there are several important contemporary biofuel types, including wood fuel and crop residue (freshly produced biomass). The $\Delta^{14}C$ for wood fuel ($\Delta^{14}C$ = 155‰) was estimated by a tree-growth model[13] including 10-year, 20-year, 40-year, 70-year, and 85-year old trees with weights of 0.2, 0.2, 0.4, 0.1, and 0.1, respectively, logged in the 2010s. The $\Delta^{14}C$ for cope residue corresponds to contemporary $^{14}CO_2$ in year 2016 ($\Delta^{14}C$=15‰). To regionally parameterize the contemporary $\Delta^{14}C_{\mathrm{bb}}$ end member, the relative contribution of fuel wood (57%) and crop residue (43%) provided by Tao et al.[14] were employed. Hence, a China-tailored $\Delta^{14}C_{\mathrm{bb}}$ of 95‰ ($\Delta^{14}C_{\mathrm{bb}}$ = 155‰×0.57 + 15‰×0.43) was used. The corresponding $F_{m,\ bio}$ and $F_{m,\ bb}$ values were 1.02 and 1.10, respectively, calculated by the following equation:

$$\Delta^{14}C = \left(Fm \times e^{\lambda(1950-x)} - 1\right) \times 1000‰ \qquad \textbf{Eq.S8}$$

where λ is 1/(true mean-life) of radiocarbon (corresponding to 1/8267) and x is the year of collection (in this case 2018).The $F_{m,\mathrm{ref}}$ were estimated as 1.06 (i.e., $F_{m,\mathrm{ref}}$ = 1.10× 0.5 + 1.02×0.5), based on the assumption that contemporary carbon originates equally from biogenic emission and biomass burning[13].

Supplementary Method 4. $\delta^{13}C$ and $\Delta^{14}C$ analysis of water-soluble organic carbon

Filters were extracted for water-soluble organic carbon (WSOC) in 15mL ultrapure water under ultrasonication for three times, and then the water extracts were filtered through a 0.22-μm PTFE membrane to remove insoluble particles. About 15mL of WSOC extraction were frozen at -20°C and completely dried in a vacuum freeze drier. The residue was redissolved in ~200 μL ultrapure water. Each sample was divided into two capsules: 50 μL was transferred into tin capsule for stable carbon isotopic measurements and the rest into capsule for radiocarbon measurements. The concentrated samples were completely evaporated in oven at 60°C before isotopic analyses.

Determination of the carbon isotopes of WSOC composition follows the previously published procedure[15]. The $\delta^{13}C$ of WSOC were measured using a Flash 2000 elemental analyzer connected to a Delta V IRMS. The $\Delta^{14}C$ composition of WSOC were measured at the accelerator mass spectrometry (AMS) facility of the Guangzhou Institute of Geochemistry of the Chinese Academy of Sciences (GIGCAS). In general, more than 200 μg of WSOC were combusted and converted to graphite for radiocarbon analysis. A total of 200 μg of C of radiocarbon-dead blank material (phthalic anhydride, Sigma-Aldrich) or standards (IAEA C7 oxalic acid) were added to the tin capsule alongside the dried WSOC. All fraction modern values of WSOC reported have been corrected for combustion and graphitization. The Fm values of WSOC was converted to the fraction of non-fossil source as discussed in Supplementary Method 3.

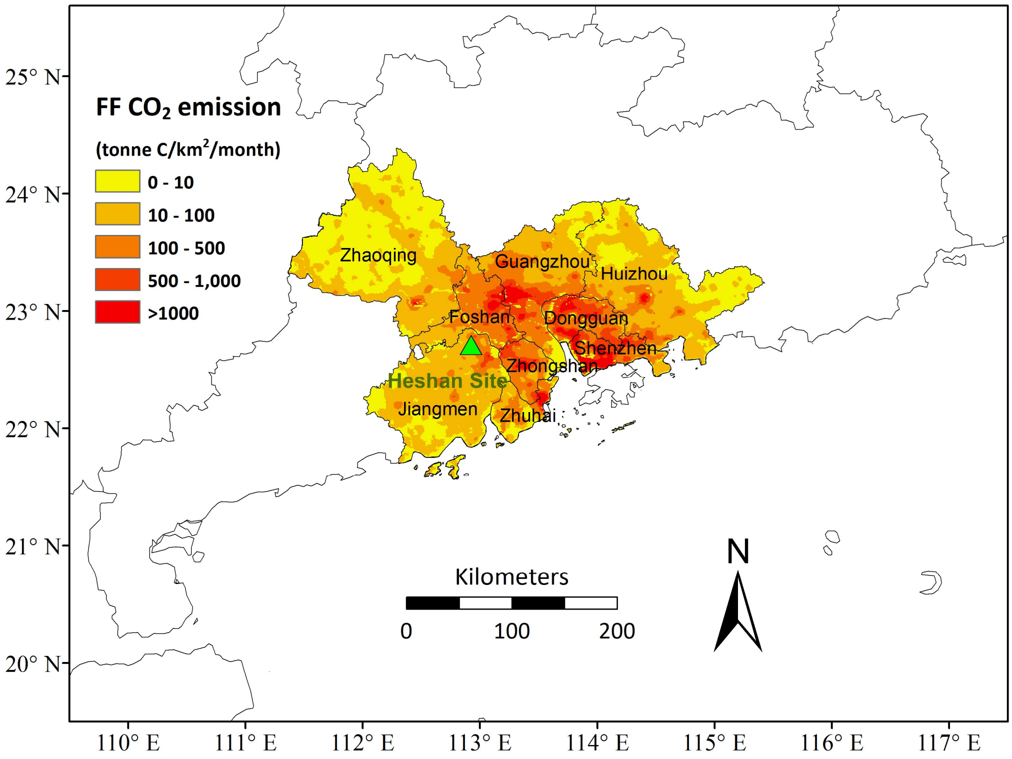

Supplementary Figure 1. The average fossil fuel CO_2 (FF CO_2) emissions during June 2017 to May 2018 over Pearl River Delta (PRD) with indication of the location of the Heshan receptor site in the southwest of the PRD. The FF CO_2 data is obtained from Open-source Data Inventory for Anthropogenic CO_2 (https://db.cger.nies.go.jp/dataset/ODIAC/DL_odiac2020b.html). The geographic boundaries in the map are originated from DataV.GeoAtlas free vector map data (http://datav.aliyun.com/portal/school/atlas/area_selector).

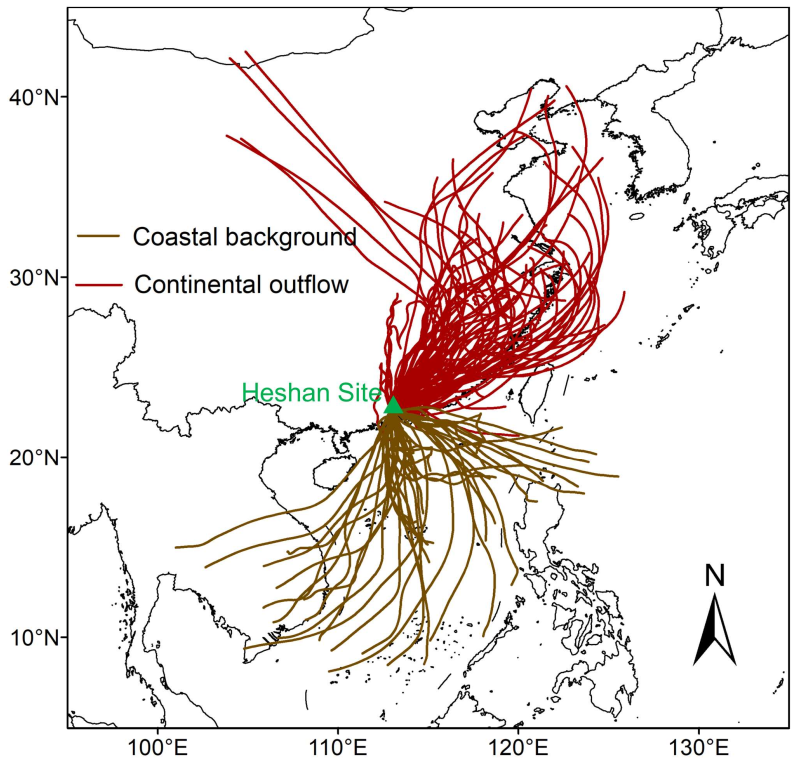

Supplementary Figure 2. Three-day backward trajectories (BTs) arriving at an altitude of 100 m over Heshan for every 6h. Based on BTs, aerosol samples are categorized into two major transport pathways: Coastal background and Continental outflow. The coastline boundaries in the map are originated from Natural Earth free vector map data (https://www.naturalearthdata.com/). The administration boundaries in the map are originated from map products of National Geomatics Center of China (https://www.webmap.cn/).

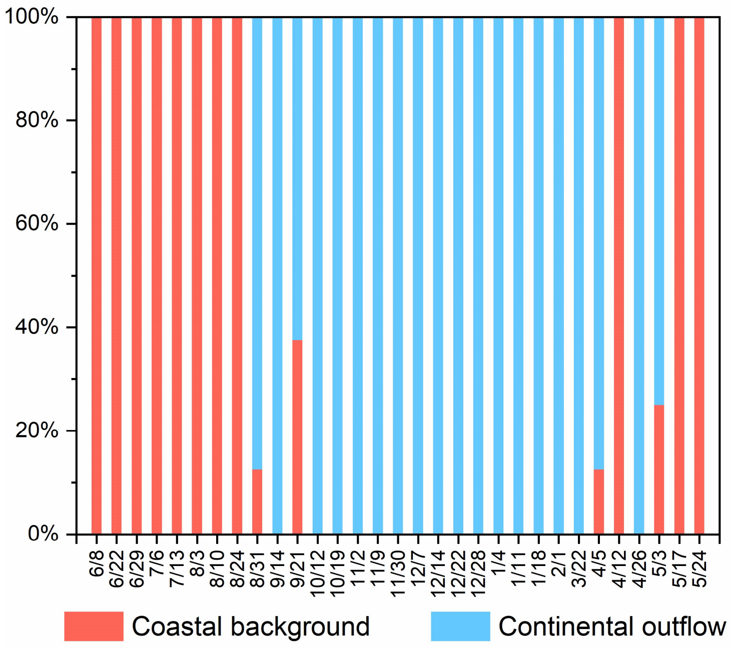

Supplementary Figure 3. Fractional contribution of air mass clusters in each aerosol sample during the sampling campaign (Jun 2017 to May 2018). Red column represents the contribution of air mass originate from the South China Sea (coastal background), whereas blue column represents the contribution of air mass originate from Chinese continental (continental outflow).

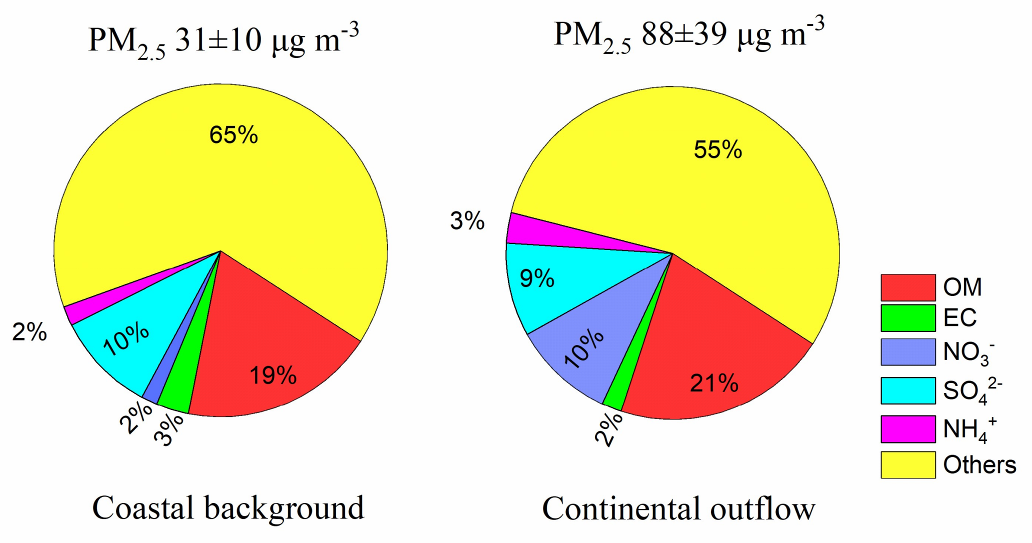

Supplementary Figure 4. Organic matter (OM), elemental carbon (EC), and water-soluble inorganic constituents (WSIC) in their total $PM_{2.5}$ concentration.

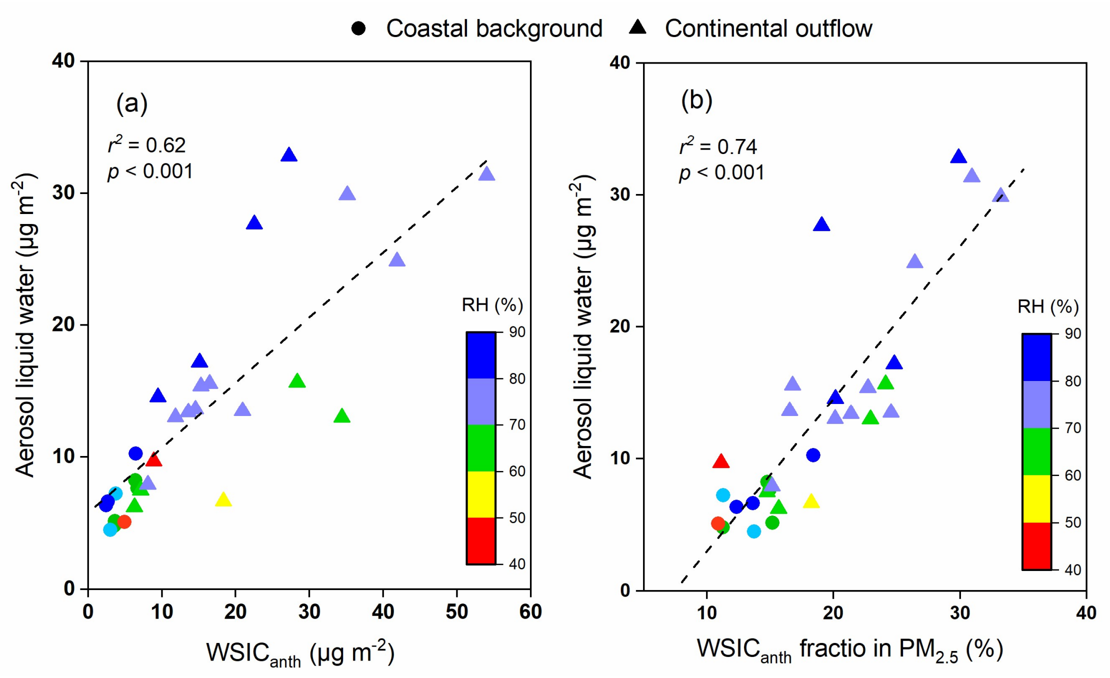

Supplementary Figure 5. Relationships between (a) aerosol liquid water content and anthropogenic water-soluble inorganic constituents ($WSIC_{anth}$); (b) aerosol liquid water content and $WSIC_{anth}$ to $PM_{2.5}$ mass ratio.

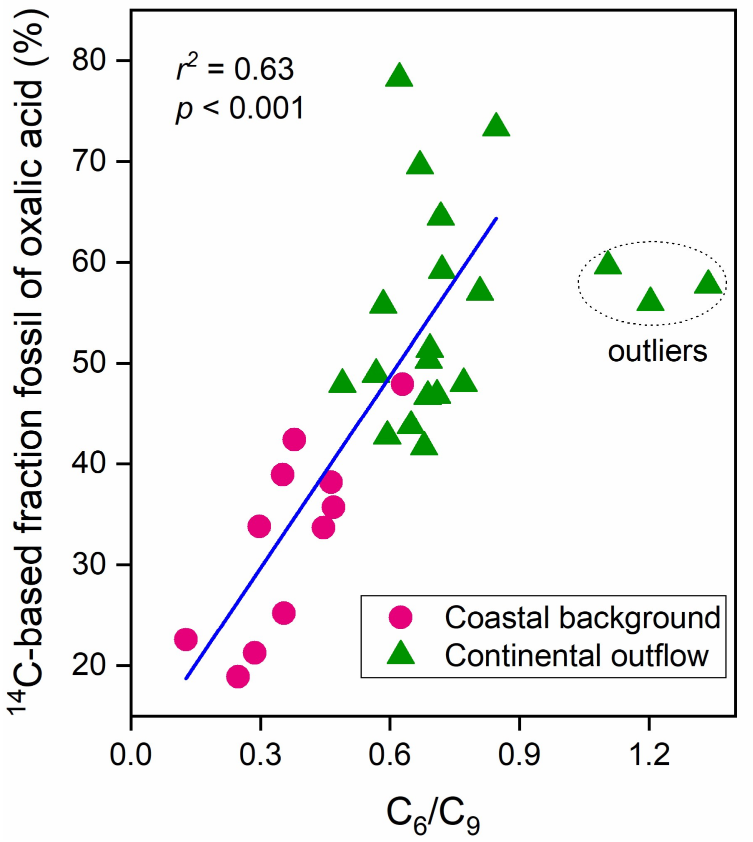

Supplementary Figure 6. Relationships between fraction fossil of oxalic acid versus adipic to azelaic acid (C_6/C_9) ratio.

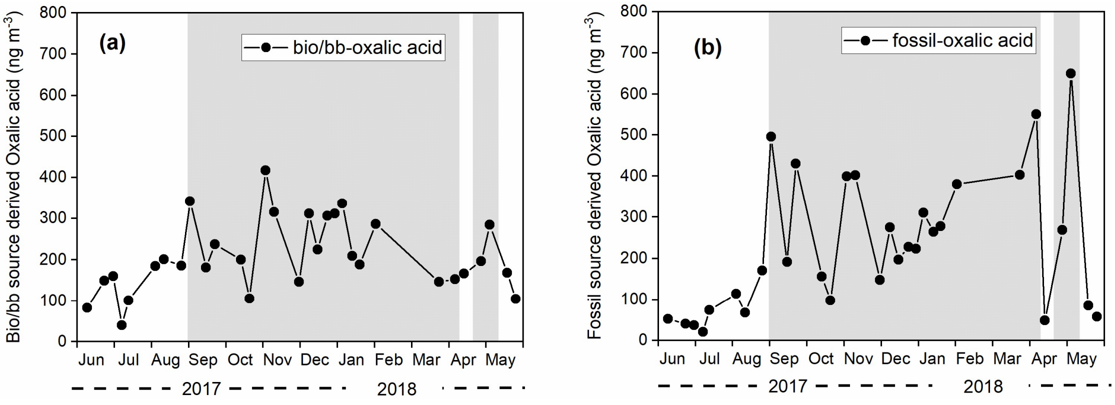

Supplementary Figure 7. Radiocarbon-based concentration of oxalic acid from (a) biogenic/biomass burning (bio/bb) and (b) fossil sources over Heshan from 2017 to 2018. The unshaded periods represent coastal background air mass regime, while the shadowed periods represent continental outflow air mass regime.

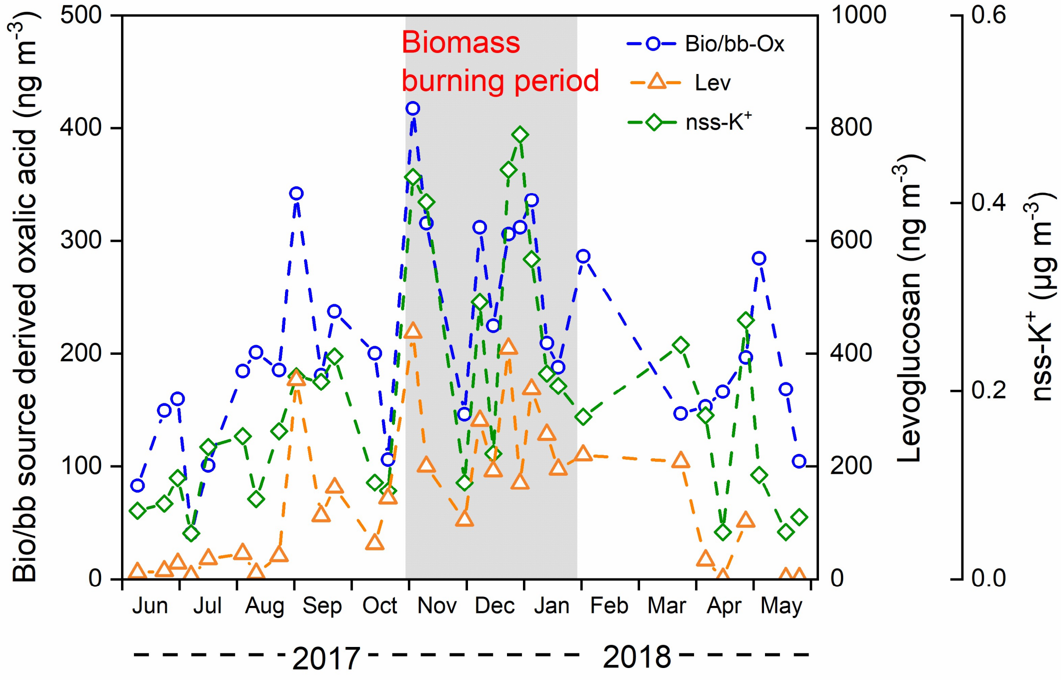

Supplementary Figure 8. Temporal variation of biogenic/biomass burning (bio/bb) source derived oxalic acid (bio/bb-Ox), non-sea salt K^+ (nss-K^+), and Levoglucosan (Lev) for the $PM_{2.5}$ collected in Heshan from Jun 2017 to May 2018. The shadows represent the period from November to February when open burning of post-harvest agricultural crop residues is a widespread phenomenon.

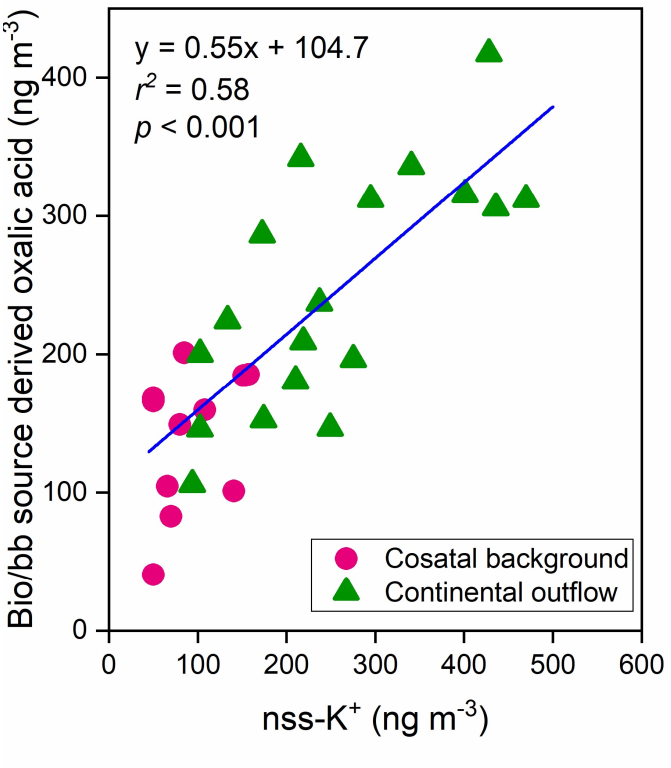

Supplementary Figure 9. Relationships between biogenic/biomass burning (bio/bb) source derived oxalic acid source derived oxalic acid versus non-sea salt K^+ (nss-K^+).

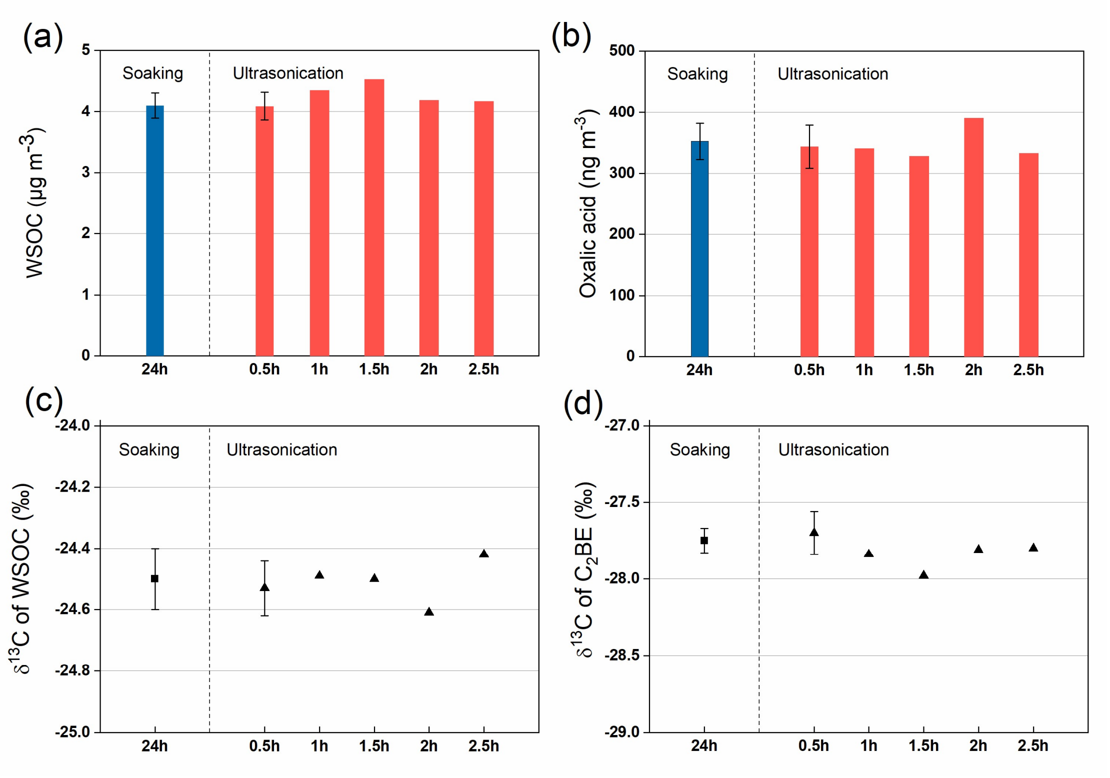

Supplementary Figure 10. Tests for the extraction protocols. (a) concentration of water-soluble organic carbon (WSOC) after soaking or ultrasonication extraction. (b) concentration of oxalic acid after soaking or ultrasonication extraction. (c) $\delta^{13}C$ composition of WSOC after soaking or ultrasonication extraction. (d) $\delta^{13}C$ composition of oxalic acid dibutyl esters (C_2BE) after soaking or ultrasonication extraction. The error bars represent standard deviation from three replicate experiments. Error bars represent the standard error of the mean with the number of replicates (n = 3).

Supplementary Table 1. Description of the air mass origins of the aerosol samples collected at Heshan receptor site.

Characteristic	Air mass Origin	Start date	Stop date
Coastal background	South China Sea - Peral River Delta	6/8/2017	6/10/2017
		6/22/2017	6/24/2017
		6/29/2017	7/1/2017
		7/6/2017	7/8/2017
		7/13/2017	7/15/2017
		8/3/2017	8/5/2017
		8/10/2017	8/12/2017
		8/24/2017	8/26/2017
		4/12/2018	4/14/2018
		5/17/2018	5/19/2018
		5/24/2018	5/26/2018
Continental outflow	Chinese continental - Peral River Delta	8/31/2017	9/2/2017
		9/14/2017	9/16/2017
		9/21/2017	9/23/2017
		10/12/2017	10/14/2017
		10/19/2017	10/21/2017
		11/2/2017	11/4/2017
		11/9/2017	11/11/2017
		11/30/2017	12/2/2017
		12/7/2017	12/9/2017
		12/14/2017	12/16/2017
		12/22/2017	12/24/2017
		12/28/2017	12/30/2017
		1/4/2018	1/6/2018
		1/11/2018	1/13/2018
		1/18/2018	1/20/2018
		1/25/2018	1/27/2018
		2/1/2018	2/3/2018
		3/22/2018	3/24/2018
		4/5/2018	4/7/2018
		4/26/2018	4/28/2018
		5/3/2018	5/5/2018

Supplementary Table 2. Statistically description (range, mean, and standard deviation) and significant differences/similarities for meteorological parameters, and concentrations of gaseous pollutants and major chemical components between coastal background and continental outflow sampling campaign.

	Coastal background (N = 11)		Continental outflow (N = 21)		
	min-max	Mean/SD	min-max	Mean/SD	t-score, df, p-value[a]
I. Meteorological parameters					
Temperature, °C	26.3-30.2	28.4±1.2	9.1-29.1	20.2±5.5	
Relative humidity, %	68.9-89.9	82.4±6.2	56.9-85.2	74.4±9.0	
Visibility, km	12.3-39.5	26±9.3	1.6-33.4	14.2±9.5	
Wind speed, km h^{-1}	1.9-3.8	2.7±0.5	1.4-7.3	3.5±1.7	
AQI[b]	25.3-54.9	31.9±8.2	35.3-198	86.7±44.9	
II. Gaseous pollutants, μg m^{-3}					
SO_2	8.1-18.2	13.8±3.1	13.4-43.4	24.9±8.3	-4.2, 30, **<0.05**
NO_2	8.0-23.7	14.6±6.1	17.3-108.9	54.4±25.2	-5.1,30, **<0.05**
NO	1.8-13.2	6.1±4.2	2.8-22.6	9.3±6.3	-1.5, 30, >0.05
O_3	19.3-49.9	33.9±8	20.8-117	62.3±25.7	-2.8, 30, **<0.05**
CO	0.4-0.6	0.5±0.1	0.6-1.3	0.9±0.2	-5.9, 30, **<0.05**
III. Major components of $PM_{2.5}$, μg m^{-3}					
$PM_{2.5}$	19.6-45.2	31.1±10	35.7-174.7	87.8±39.3	-4.7, 30, **<0.05**
OC	2.2-5.7	3.3±1.1	4-24.3	10.9±5.9	-4.2, 30, **<0.05**
EC	0.6-1.3	0.9±0.3	1.1-2.9	1.7±0.6	-4.7, 30, **<0.05**
ALW	4.5-10.2	6.9±2.0	6.2-31.4	16.6±8.1	-3.8, 30, **<0.05**
WSOC	0.7-1.6	1.2±0.3	1.9-9.6	4.5±1.8	-5.9, 29, **<0.05**
WSON	0.00-0.60	0.28±0.21	0.1-4.3	1.4±1.0	-3.6, 29, **<0.05**
NO_3^-	0.1-1.7	0.5±0.5	0.4-34.1	8.7±8.5	-3.2, 30, **<0.05**
SO_4^{2-}	1.8-4.7	3.1±1	3.3-13.2	8.0±3.0	-5.3, 30, **<0.05**
NH_4^+	0.4-1	0.6±0.2	0.9-6.7	2.6±1.5	-4.3, 30, **<0.05**
nss-K^+	0.05-0.16	0.09±0.04	0.09-0.47	0.25±0.13	-3.9, 30, **<0.05**
IV. Mass ratios of major components of $PM_{2.5}$					
OC/EC	2.6-4.7	3.7±0.7	3.2-10.5	6±2	-3.7, 30, **<0.05**
WSOC/OC	0.3-0.6	0.4±0.1	0.3-0.8	0.5±0.1	-1.4, 29, >0.05

[a] Statistically significant differences/similarities between these two datasets based on the Unpaired Two-tailed t-Test at 95% Confidence Interval. df = degrees of freedom

[b] Air Quality Index (AQI)

Supplementary Table 3. Mass concentration of dicarboxylic acids, oxocarboxylic acids, and α-dicarbonyls as well as the concentration of $PM_{2.5}$, organic carbon (OC), elemental carbon (EC) and water-soluble organic carbon (WSOC) in coastal background and continental outflow aerosol samples.

	Costal background (N = 11)		Continental outflow (N = 21)	
	Range	Mean/SD	Range	Mean/SD
I. Dicarboxylic Acids, ng m^{-3}				
Oxalic, C_2	61.1-355.9	210.3±81.4	203.4-934.4	569.4±187.4
Malonic, C_3	2.3-22.4	13.7±5.2	13.0-50.1	33.1±9.7
Succinic, C_4	1.8-18.6	9.5±4.8	13.2-62.8	36.7±13.0
Glutaric, C_5	0.5-5.0	2.1±1.2	3.8-17.1	10.5±4.0
Adipic, C_6	0.5-3.5	1.4±0.8	2.9-8.8	5.5±1.7
Pimelic, C_7	0.2-0.9	0.5±0.2	0.8-3.4	1.7±0.7
Suberic, C_8	0.4-1.6	1.0±0.4	1.4-4.7	2.6±1.0
Azelaic, C_9	1.7-7.7	4.0±1.7	4.8-14.1	7.4±2.7
Sebacic, C_{10}	0.2-0.7	0.4±0.2	0.6-1.9	1.0±0.4
Undecanedioic, C_{11}	0.1-0.4	0.3±0.1	0.4-1.3	0.6±0.3
Dodecanedioic, C_{12}	0.0-0.2	0.2±0.1	0.2-0.7	0.3±0.1
Methylmalonic, iC_4	0.0-0.6	0.3±0.2	0.4-1.3	0.7±0.2
Methylsuccinic, iC_5	0.2-1.6	0.8±0.4	1.4-5.3	2.8±0.9
2-Methylglutaric, iC_6	0.1-0.5	0.3±0.1	0.5-1.4	0.9±0.2
Maleic, M	0.2-1.6	0.7±0.4	0.6-3.9	1.7±1.0
Fumaric, F	0.2-1.4	0.8±0.3	0.2-2.3	1.1±0.7
Methylmaleic, mM	0.3-5.0	1.5±1.2	1.0-7.4	3.1±1.8
Malic acid, hC_4	0.0-3.5	1.3±1.2	0.0-8.1	1.8±1.9
Citric acid, Cit	0.2-3.8	1.9±1.0	0.2-5.6	1.6±1.4
Phthalic, Ph	0.0-4.5	1.7±1.6	0.0-23.1	6.3±6.2
Isophthalic, iPh	0.0-0.7	0.3±0.2	0.0-4.2	1.5±1.1
Terephthalic, tPh	2.3-16.2	9.4±4.9	1.8-68.1	32.4±19.5
4-Ketopimelic, kC_7	0.2-3.8	1.3±1.0	0.0-7.9	3.3±2.2
Total diacids	74.5-452.0	263.7±100.4	307.3-1094.4	726.0±205.4
II. Oxocarboxylic acids, ng m^{-3}				
Pyruvic, Pyr	1.7-16.8	8.8±4.5	2.7-38.2	13.4±9.7
Glyoxylic, ωC_2	5.8-99.7	45.9±24.4	66.6-278.3	128.9±51.0
3-Oxopropanoic, ωC_3	0.7-6.7	3.3±1.8	0.3-7.9	2.1±2.0
4-Oxobutaboic, ωC_4	1.1-5.7	3.7±1.6	0.9-10.2	4.5±2.8
5-Oxopentanoic, ωC_5	0.2-1.0	0.6±0.3	0.3-22.2	4.4±6.2
7-Oxoheptanoic, ωC_7	0.2-2.3	1.4±0.8	0.6-4.9	1.7±1.2
8−Oxooctanoic, ωC_8	0.2-4.8	1.8±1.3	0.2-7.0	1.8±1.8
9-Oxononanoic, ωC_9	0.1-0.9	0.4±0.3	0.0-1.5	0.5±0.4
Total oxoacids	10.5-128.1	65.9±32.4	77.1-343.6	157.3±63
III. α-Dicarbonyls, ng m^{-3}				
Glyoxal, Gly	0.2-4.6	1.5±1.3	0.5-36.8	4.9±7.9
Methylglyoxal, MeGly	0.8-72.0	19.4±24.2	12.9-532.3	169.3±173.9

Total dicarbonyls	1.0-75.1	20.9±25.4	14.5-543.7	174.2±178.0
$PM_{2.5}$, μg m^{-3}	19.6-45.2	31.1±10.0	35.7-174.4	87.8±39.3
OC, μg m^{-3}	2.2-5.7	3.3±1.1	4.0-24.3	10.9±5.9
EC, μg m^{-3}	0.6-1.3	0.9±0.3	1.1-2.9	1.7±0.6
WSOC, μg m^{-3}	0.7-1.6	1.2±0.3	1.9-9.6	4.5±1.8

Supplementary Table 4. Diagnostic mean mass ratios of dicarboxylic acids and related compounds in coastal background and continental outflow aerosol samples. For abbreviation, see Supplementary Table 3.

	Coastal background	Continental outflow
C_2/total diacids (%)	79.7 ± 4.0	77.4 ± 6.4
C_3/total diacids (%)	5.2 ± 1.4	4.7 ± 1.4
C_4/total diacids (%)	3.5 ± 0.8	5.1 ± 1.4
C_6/C_9	0.37 ± 0.13	0.76 ± 0.21
Ph/C_9	0.43± 0.35	0.84 ± 0.68
C_3/C_4	1.5 ± 0.36	0.94 ± 0.25
M/F	0.94 ± 0.23	1.9 ± 0.92
dicarboxyl-C/OC (%)	0.2 ± 0.2	1.3 ± 1.5
WSOC/OC (%)	39.1 ±10.5	44.0 ±11.8

Supplementary Table 5. Stable carbon isotope ratios ($\delta^{13}C$, ‰) of major sources of biogenic VOCS (BVOCs) and anthropogenic VOCs (AVOCs).

	Source	Compounds	$\delta^{13}C$ (‰)
BVOCs	Myrtle, buckthrom, velvet bean[a]	Isoprene	-29.2‰ ± 0.6‰
	velvet bean[b]	Isoprene	-27.7‰ ± 2‰
	Mixed deciduous forest[c]	Isoprene	-29‰ to -26‰
	44 C3 plants[d]	Terpenoids	-27.1‰ ± 2.5‰
AVOCs	Industrial stack[e]	Benzene and Toluene	-25.4‰ to -23.5‰
	Industrial stack[f]	*n*-alkanes, aromatics, ketones and *n*-alcohol	-25.5‰ ± 2.5‰
	Coal[g]	2-6 rings aromatics	-29.0‰ to -24.2‰
	Biomass burning (C3 plants)[h]	Non-methane Hydrocarbons	-26.5‰ to -25.7‰
	Biomass burning (C3 plants)[i]	Benzene and Toluene	-27.6‰ to -27.1‰
	Biomass burning (C3 plants)[j]	C_6-C_{10} VOCs	-27.6‰ ± 1.6‰
	Transportation[k]	Non-methane Hydrocarbons	-27.7‰ ± 1.7‰
	Fossil fuel combustion[i]	Benzene and Toluene	-27.5‰ to -26.9‰
	Gas station[l]	C_5-C_{11} VOCs	-26.8‰ ± 1.9‰
	Gasoline[m]	Non-methane Hydrocarbons	-26.4‰ ± 1.1‰

[a] Affek and Yakir (2003)[16]. [b] Rudolph et al. (2003)[17]. [c] Iannone et al. (2007)[18]. [d] Diefendorf et al. (2012)[19]. [e] Turner et al. (2006)[20]. [f] Vitzthum et al. (2011)[21]. [g] McRae et al. (1996)[22]. [h] Czapiewski et al. (2002)[23]. [i] Giebel et al. (2010)[24]. [j] Vitzthum et al. (2012)[25]. [l] Kawashima et al. (2014)[26]. [m] Averages and standard deviations was estimated by a figure reported by Smallwood et al. (2002)[27].

Supplementary Table 6. Compound-specific stable carbon isotope ratios ($\delta^{13}C$, ‰) of dicarboxylic acid and oxocarboxylic acids along with their statistical significance between coastal background samples and continental outflow samples.

	Coastal background (N = 11)		Continental outflow (N=21)		
	Range	Mean/SD	Range	Mean/SD	t-score, df, p-value [a]
I. Dicarboxylic Acids					
Oxalic, C_2	-23.3 to -14.6	-19.9±2.3	-30.7 to -21.3	-24.6±2.7	4.9, 29, **< 0.05**
Malonic, C_3	-34.3 to -21.9	-27.3±3.2	-36.6 to -24.8	-29.1±3.7	1.3, 29, > 0.05
Succinic, C_4	-33.6 to -26.7	-29.2±2.1	-35.4 to -26.5	-30.5±2.2	1.6, 29, > 0.05
Glutaric, C_5	-40.8 to -27.9	-34.0±4.4	-41.9 to -25.7	-33.4±4.7	-0.3, 25, > 0.05
Azelaic, C_9	-40.7 to -30.8	-34.1±2.8	-38 to -28.1	-34.7±2.6	0.6, 28, > 0.05
Phthalic, Ph	-43.9 to -29.5	-34.1±4.0	-37 to -29.1	-33.2±3.2	-0.5, 15, > 0.05
Terephthalic, tPh	-32.6 to -27.3	-29.4±1.8	-36.9 to -25.5	-28.7±3.1	-0.7, 21, > 0.05
II. Oxocarboxylic acids					
Glyoxylic, ωC_2	-36.3 to -25.0	-30.4±4.1	-43.8 to -24.1	-34.8±4.5	2.6, 29, **< 0.05**
Pyruvic, Pyr	-32.8 to -22.1	-27.7±3.1	-37.2 to -22.7	-30.9±4.5	2.0, 18, **< 0.05**

[a] Statistically significant differences/similarities between these two datasets based on the Unpaired Two-tailed t-Test at 95% Confidence Interval. df = degrees of freedom

Supplementary Table 7. Radiocarbon isotopic analyses of oxalic acid in coastal background samples and continental outflow samples.

	Sampling date	Size (μg C)	F_m	$f_{bio/bb-C2}$ (%)	$f_{fossil-C2}$ (%)
Coastal background	8-10 Jun	90	0.65±0.03	61.1±2.8	38.9±2.8
	22-24 Jun	147	0.83±0.02	78.7±1.9	21.3±1.9
	29 Jun-1 Jul	163	0.86±0.02	81.1±1.6	18.9±1.6
	6-8 Jul	30	0.70±0.09	66.2±8.5	33.8±8.5
	13-15 Jul	199	0.61±0.01	57.6±1.4	42.4±1.4
	3-5 Aug	65	0.66±0.04	61.8±3.9	38.2±3.9
	10-12 Aug	27	0.79±0.1	74.8±9.4	25.2±9.4
	24-26 Aug	188	0.55±0.02	52.1±1.5	47.9±1.5
	12-14 Apr	163	0.82±0.02	77.4±1.7	22.6±1.7
	17-19 May	56	0.70±0.05	66.3±4.5	33.7±4.5
	24-26 May	35	0.68±0.08	64.3±7.3	35.7±7.3
	**Average**		**0.71±0.1**	**67.4±9.4**	**32.6±9.4**
Continental outflow	31 Aug-2 Sep	351	0.43±0.01	40.8±0.9	59.2±0.9
	14-16 Sep	266	0.52±0.01	48.6±1.1	51.4±1.1
	21-23 Sep	274	0.38±0.01	35.5±1	64.5±1
	12-14 Oct	110	0.6±0.03	56.2±2.4	43.8±2.4
	19-21 Oct	153	0.55±0.02	52.1±1.8	47.9±1.8
	2-4 Nov	154	0.54±0.02	51.1±1.7	48.9±1.7
	9-11 Nov	191	0.47±0.02	44±1.4	56±1.4
	30 Nov-2 Dec	190	0.53±0.02	49.7±1.5	50.3±1.5
	7-9 Dec	268	0.56±0.01	53.2±1.1	46.8±1.1
	14-16 Dec	54	0.56±0.05	53.3±4.8	46.7±4.8
	22-24 Dec	353	0.61±0.01	57.3±0.9	42.7±0.9
	28-30 Dec	238	0.62±0.01	58.3±1.2	41.7±1.2
	4-6 Jan	109	0.55±0.03	52±2.4	48±2.4
	11-13 Jan	222	0.47±0.01	44.2±1.2	55.8±1.2
	18-20 Jan	91	0.43±0.03	40.4±2.9	59.6±2.9
	25-27 Jan	nd	nd	nd	nd
	1-3 Feb	156	0.46±0.02	43±1.7	57±1.7
	22-24 Mar	67	0.28±0.04	26.7±4	73.3±4
	6-8 Apr	104	0.23±0.03	21.7±2.5	78.3±2.5
	26-28 Apr	101	0.45±0.03	42.3±2.6	57.7±2.6
	3-5 May	181	0.32±0.02	30.4±1.5	69.6±1.5
	**Average**		**0.48±0.11**	**45.0±10.2**	**55.0±10.2**

Supplementary Table 8. Radiocarbon isotopic analyses of malonic acid (C_3), succinic acid (C_4), glyoxylic acid (ωC_2), and methylglyoxal (MeGly) in continental outflow samples.

Month	Compounds	Size (μg C)	F_m	$f_{bio/bb}$ (%)
September	C_3	128	0.79±0.02	74.9±1.8
	C_4	144	0.7±0.01	65.9±1.1
	ωC_2	80	0.35±0.03	32.7±2.6
November	C_3	50	0.71±0.05	66.7±4.3
	C_4	nd	nd	nd
	ωC_2	80	0.33±0.03	30.8±2.6
December	C_3	156	0.72±0.02	68±2.2
	C_4	210	0.65±0.01	61.7±0.9
	ωC_2	82	0.37±0.03	35.1±2.6
January	C_3	74	0.69±0.04	65.3±3.8
	C_4	132	0.69±0.01	65.5±1.1
	ωC_2	110	0.27±0.01	25.7±1.3
	MeGly	119	0.34±0.05	32.5±4.6

Supplementary Table 9. The stable carbon ($\delta^{13}C$) and radiocarbon (F_m) composition of water-soluble organic carbon (WSOC) and oxalic acid (C_2) in ambient aerosols in the five major emission hot spot megacities of China.

City	Location	Season	$\delta^{13}C$-WSOC (‰)	$\delta^{13}C$-C_2 (‰)	F_m-WSOC	F_m-C_2
Guangzhou	23.149°N, 113.358°E	Summer	-25.2±0.02	-23.2±0.25	0.657±0.002	0.77±0.026
		Winter	-24.9±0.05	-27.5±0.35	0.707±0.003	0.675±0.015
Beijing	39.974°N, 116.370°E	Summer	-24.3±0.1	-23.7±0	0.476±0.002	0.461±0.015
		Winter	-24.4±0.12	-29.7±1.06	0.633±0.002	0.467±0.052
Wuhan	30.531°N, 114.308°E	Summer	-25±0.07	-22.6±0.25	0.709±0.003	0.775±0.047
		Winter	-23.8±0.02	-27.2±0.6	0.639±0.002	0.632±0.014
Chengdu	30.629°N, 104.064°E	Summer	-25.9±0.06	-25.1±0.88	0.659±0.004	0.666±0.048
		Winter	-23.8±0.09	-26.6±1.2	0.834±0.002	0.629±0.01
Shanghai	31.316°N, 121.423°E	Summer	-24.1±0.11	-18.6±0.18	0.663±0.003	0.759±0.016
		Winter	-24.3±0.12	-24.7±0.95	0.52±0.003	0.42±0.012

Supplementary Table 10. Comparison of soaking and ultrasonication method

	Soaking-24 h [a]				Ultrasonic-30 min [a]				Ultrasonic -60 min	Ultrasonic -90 min	Ultrasonic -120 min	Ultrasonic -150 min
	first	second	third	av	first	second	third	av				
WSOC (μg m^{-3})	3.86	4.18	4.25	4.10 ± 0.21	4.35	4.02	3.90	4.09 ± 0.23	4.35	4.53	4.19	4.17
$\delta^{13}C$-WSOC (‰)	-24.44	-24.62	-24.43	-24.50 ± 0.10	-24.43	-24.59	-24.59	-24.53 ± 0.09	-24.49	-24.50	-24.61	-24.42
C_2 (ng m^{-3})	384.8	344.1	328.0	352.3 ± 29.3	304.3	373.0	353.7	343.7 ±35.4	340.4	328.0	390.4	333.0
$\delta^{13}C$-C_2BE [b] (‰)	-27.84	-27.70	-27.71	-27.75 ± 0.08	-27.87	-27.67	-27.60	-27.70 ± 0.14	-27.84	-27.98	-27.81	-27.80

a. three replicate experiments were performed. b. oxalic acid dibutyl esters

Supplementary References

1. Mo Y*, et al.* Sources, compositions, and optical properties of humic-like substances in Beijing during the 2014 APEC summit: Results from dual carbon isotope and Fourier-transform ion cyclotron resonance mass spectrometry analyses. *Environ. Pollut.* **239**, 322-331 (2018).
2. Fu P, Kawamura K, Kanaya Y, Wang Z. Contributions of biogenic volatile organic compounds to the formation of secondary organic aerosols over Mt. Tai, Central East China. *Atmos. Environ.* **44**, 4817-4826 (2010).
3. Fu P*, et al.* Organic molecular compositions and temporal variations of summertime mountain aerosols over Mt. Tai, North China Plain. *J. Geophys. Res.* **113**, (2008).
4. Fountoukis C, Nenes A. ISORROPIA II: a computationally efficient thermodynamic equilibrium model for K+-Ca2+-Mg2+-Nh(4)(+)-Na+-SO42--NO3--Cl--H2O aerosols. *Atmos. Chem. Phys.* **7**, 4639-4659 (2007).
5. Nguyen TKV, Zhang Q, Jimenez JL, Pike M, Carlton AG. Liquid water: ubiquitous contributor to aerosol mass. *Environ. Sci. Technol. Lett.* **3**, 257-263 (2016).
6. Gong Z*, et al.* Characterization of submicron aerosols in the urban outflow of the central Pearl River Delta region of China. *Front. Environ. Sci. Eng.* **6**, 725-733 (2012).
7. Kreidenweis SM, Petters MD, DeMott PJ. Single-parameter estimates of aerosol water content. *Environ. Res. Lett.* **3**, (2008).
8. Kawamura K, Watanabe T. Determination of stable carbon isotopic compositions of low molecular weight dicarboxylic acids and ketocarboxylic acids in atmospheric aerosol and snow samples. *Anal. Chem.* **76**, 5762-5768 (2004).
9. Ishikawa NF*, et al.* Improved Method for Isolation and Purification of Underivatized Amino Acids for Radiocarbon Analysis. *Anal. Chem.* **90**, 12035-12041 (2018).
10. Ziolkowski LA, Druffel ER. Quantification of extraneous carbon during compound specific radiocarbon analysis of black carbon. *Anal. Chem.* **81**, 10156 (2009).
11. Gustafsson Ö*, et al.* Brown Clouds over South Asia: Biomass or Fossil Fuel Combustion. *Science* **323**, 495-498 (2009).
12. Zhang G*, et al.* Radiocarbon isotope technique as a powerful tool in tracking anthropogenic emissions of carbonaceous air pollutants and greenhouse gases: A review. *Fundam. Res.* **1**, 306-316 (2021).
13. Zhang YL*, et al.* Radiocarbon-based source apportionment of carbonaceous aerosols at a regional background site on Hainan Island, South China. *Environ. Sci. Technol.* **48**, 2651-2659 (2014).
14. Tao S*, et al.* Quantifying the rural residential energy transition in China from 1992 to 2012 through a representative national survey. *Nat. Energy* **3**, 567-573 (2018).
15. Mo Y*, et al.* Dual carbon isotope-based source apportionment and light absorption properties of water-soluble organic carbon in PM2.5 over China. *J. Geophys. Res.: Atmos.*, e2020JD033920 (2021).
16. Affek HP, Yakir D. Natural abundance carbon isotope composition of isoprene reflects incomplete coupling between isoprene synthesis and photosynthetic carbon flow. *Plant Physiol.* **131**, 1727-1736 (2003).
17. Rudolph J*, et al.* The stable carbon isotope ratio of biogenic emissions of isoprene and the

potential use of stable isotope ratio measurements to study photochemical processing of isoprene in the atmosphere. *J. Atmos. Chem.* **44**, 39-55 (2003).

18. Iannone R, Koppmann R, Rudolph J. A technique for atmospheric measurements of stable carbon isotope ratios of isoprene, methacrolein, and methyl vinyl ketone. *J. Atmos. Chem.* **58**, 181-202 (2007).
19. Diefendorf AF, Freeman KH, Wing SL. Distribution and carbon isotope patterns of diterpenoids and triterpenoids in modern temperate C3 trees and their geochemical significance. *Geochim. Cosmochim. Acta* **85**, 342-356 (2012).
20. Turner N*, et al.* δ13C of volatile organic compounds (VOCS) in airborne samples by thermal desorption-gas chromatography-isotope ratio-mass spectrometry (TD-GC-IR-MS). *Atmos. Environ.* **40**, 3381-3388 (2006).
21. Vitzthum von Eckstaedt C, Grice K, Ioppolo-Armanios M, Jones M. δ13C and δD of volatile organic compounds in an alumina industry stack emission. *Atmos. Environ.* **45**, 5477-5483 (2011).
22. McRae C, Love GD, Murray IP, Snape CE, Fallick AEJAC. Potential of gas chromatography isotope ratio mass spectrometry to source polycyclic aromatic hydrocarbon emissions. *Anal. Commun.* **33**, 331-333 (1996).
23. Czapiewski Kv*, et al.* Isotopic composition of non-methane hydrocarbons in emissions from biomass burning. *J. Atmos. Chem.* **43**, 45-60 (2002).
24. Giebel BM, Swart PK, Riemer DD. δ13C Stable Isotope Analysis of Atmospheric Oxygenated Volatile Organic Compounds by Gas Chromatography-Isotope Ratio Mass Spectrometry. *Anal. Chem.* **82**, 6797-6806 (2010).
25. Vitzthum von Eckstaedt CD, Grice K, Ioppolo-Armanios M, Kelly D, Gibberd M. Compound specific carbon and hydrogen stable isotope analyses of volatile organic compounds in various emissions of combustion processes. *Chemosphere* **89**, 1407-1413 (2012).
26. Kawashima H, Murakami M. Measurement of the stable carbon isotope ratio of atmospheric volatile organic compounds using chromatography, combustion, and isotope ratio mass spectrometry coupled with thermal desorption. *Atmos. Environ.* **89**, 140-147 (2014).
27. Smallwood BJ, Paul Philp R, Allen JD. Stable carbon isotopic composition of gasolines determined by isotope ratio monitoring gas chromatography mass spectrometry. *Org. Geochem.* **33**, 149-159 (2002).
